# A retrospective audit of antibiotic prescriptions in primary health-care facilities in Eastern Region, Ghana

**DOI:** 10.1093/heapol/czv048

**Published:** 2015-06-04

**Authors:** Mary-Anne Ahiabu, Britt P Tersbøl, Richard Biritwum, Ib C Bygbjerg, Pascal Magnussen

**Affiliations:** ^1^Disease Control and Prevention Department, Ghana Health Service, Ministry of Health, P. O. Box KB 493, Accra, Ghana,; ^2^Department of Public Health, University of Copenhagen, Øster Farimagsgade 5, P.O. Box 2099, 1014 Copenhagen K, Denmark,; ^3^Department of Community Health, College of Health Sciences, University of Ghana P. O. Box KB 4236, Accra, Ghana and; ^4^Centre for Medical Parasitology, University of Copenhagen, CSS Building 22/23, Øster Farimagsgade 5, PO Box 2099, 1014 Copenhagen K, Denmark

**Keywords:** Antibiotic use, developing country, prescribing, primary health care, rational drug use

## Abstract

Resistance to antibiotics is increasing globally and is a threat to public health. Research has demonstrated a correlation between antibiotic use and resistance development. Developing countries are the most affected by resistance because of high infectious disease burden, limited access to quality assured antibiotics and more optimal drugs and poor antibiotic use practices. The appropriate use of antibiotics to slow the pace of resistance development is crucial. The study retrospectively assessed antibiotic prescription practices in four public and private primary health-care facilities in Eastern Region, Ghana using the WHO/International Network for the Rational Use of Drugs rational drug use indicators. Using a systematic sampling procedure, 400 prescriptions were selected per facility for the period April 2010 to March 2011. Rational drug use indicators were assessed in the descriptive analysis and logistic regression was used to explore for predictors of antibiotic prescription. Average number of medicines prescribed per encounter was 4.01, and 59.9% of prescriptions had antibiotics whilst 24.2% had injections. In total, 79.2% and 88.1% of prescribed medicines were generics and from the national essential medicine list, respectively. In the multivariate analysis, health facility type (odds ratio [OR] = 2.05; 95% confidence interval [CI]: 1.42, 2.95), patient age (OR = 0.97; 95% CI: 0.97, 0.98), number of medicines on a prescription (OR = 1.85; 95% CI: 1.63, 2.10) and ‘no malaria drug’ on prescription (OR = 5.05; 95% CI: 2.08, 12.25) were associated with an antibiotic prescription. A diagnosis of upper respiratory tract infection was positively associated with antibiotic use. The level of antibiotic use varied depending on the health facility type and was generally high compared with the national average estimated in 2008. Interventions that reduce diagnostic uncertainty in illness management should be considered. The National Health Insurance Scheme, as the main purchaser of health services in Ghana, offers an opportunity that should be exploited to introduce policies in support of rational drug use.

Key Messages

- The level of antibiotic use in primary health-care facilities was high and inappropriate use is probable considering the disease profile of Ghana.
- The limited use of diagnostic tools and non-adherence to standard treatment guidelines is contributing to the high level of antibiotic use.
- The main purchaser of health services in Ghana, the National Health Insurance Scheme, is strategically positioned to introduce systems that can modernize health-care delivery in Ghana, and this potential must be exploited in support of rational drug use.

## Introduction

Increasing bacterial resistance to common antibiotics is a serious challenge to health-care systems globally (Levy and Marshall 2004; Leung *et al*. 2011). Studies from low-, middle- and high-income countries show that antibiotics are used inappropriately, a factor that correlates positively with antibiotic resistance development (Bronzwaer *et al*. 2002; van de Sande-Bruinsma *et al*. 2008; World Health Organization 2009). Several studies, especially from Africa and Asia, report inappropriate use levels >50% (World Health Organization 2009). Monitoring the use of antibiotics in countries, exploring for factors that promote the inappropriate use of antibiotics and fashioning relevant interventions are important in slowing the pace of resistance development (Radyowijati and Haak 2003; Amábile-Cuevas 2010; Leung *et al*. 2011). The World Health Organization (1993) and the International Network for the Rational Use of Drugs (INRUD) have developed standardized indicators for monitoring rational drug use, and these indicators have been used widely to assess the quality of prescribing in health delivery systems (World Health Organization 1993, 2009).

In Ghana, the Ministry of Health (MOH) occasionally conducts assessments of drug use within public health-care facilities, the latest in 2008 (Arhinful 2009). However these surveys do not assess rational drug use in private health-care facilities even though these constitute 42% of health-care providers in Ghana (World Bank 2009). Sampling of health facilities for survey is also skewed towards hospitals compared with health centres/clinics although health centres/clinics represent >80% of service centres (Arhinful 2009; Ghana Health Service 2010). Drug use may be influenced by patient, prescriber, health facility and health system related factors. Although Ghana is undergoing an epidemiologic transition with a reduction in infectious diseases and an increase in non-communicable diseases (Institute for Health Metrics and Evaluation 2013), the five most common diseases seen among outpatients in 2008 were malaria (47.4%), upper respiratory tract infections (URTIs) (7.2%), skin diseases (4.2%), diarrhoea (3.6%) and hypertension (3.0%) (Ghana Health Service 2010). Prescribing in Ghana is conducted by physicians and non-physicians such as medical assistants (post-secondary training in diagnostics and therapeutics), nurses and other paramedics (Arhinful 2009). To guide prescribing practice, the MOH in Ghana has developed an essential drug list and therapeutic guidelines, first published in 1988 and revised every 3–5 years (Ministry of Health 2010a). Other guidelines for the integrated management of childhood illnesses, malaria, tuberculosis and HIV/AIDS exist (Baiden *et al*. 2011). However, the adoption of these guidelines by prescribers is voluntary. Changes that may influence prescribing practices, since the MOH assessment of medicine use in 2008, include (1) a revision of the standard treatment guidelines in 2010; (2) a 23% improvement in the doctor–population ratio between 2008 and 2011 and (3) a sustained steep growth in health facility utilization (42.6% between 2005 and 2008 and 39.0% between 2008 and 2011) following the operationalization of the National Health Insurance Scheme (NHIS) in 2005 leading to exponential increases in number of patients seeking care from health-care facilities (Ghana Health Service 2010, 2012a). Several reports suggest an increasing prevalence of antibiotic resistance in Ghana which may inform prescriber practices (Mills-Robertson *et al*. 2003; Donkor and Nartey 2008; Groß *et al*. 2011; Namboodiri *et al*. 2011). Health insurance coverage rate among outpatients in Ghana is high (82%) although at the population level, it is 35.2% (National Health Insurance Authority n.d.; Ghana Health Service 2012a). The NHIS provides full health-care coverage for ∼95% of diseases that afflict the population and members can access care from both public and private health-care facilities accredited by the National Health Insurance Authority (n.d.). All essential medicines, except those for managing diseases not covered by the NHIS, are reimbursable under the scheme (National Health Insurance Authority 2011).

As the health system in Ghana and other low-/middle-income countries evolves in an attempt to achieve universal health-care coverage, the need to monitor the quality of health care in this context is crucial and rational drug use is a quality indicator that can be measured using routine health facility data (Jong-wook 2003; Sambo *et al*. 2013). Although health insurance schemes often results in increased health service utilization (Blanchet *et al*. 2012, Ghana Health Service 2012a), the effect on quality of health care is often unclear (Spaan *et al*. 2012; Robyn *et al*. 2013). However, high patient load is known to promote inappropriate prescription practices (Radyowijati and Haak 2003; Das and Das 2006). Methods of reimbursement of drug cost may also influence prescribing practices (Hulscher *et al*. 2010). The fee-for-service approach (i.e. itemized billing of drugs dispensed), which is used in Ghana, may encourage health facilities to prescribe liberally to increase revenue. The NHIS, in 2011, piloted a capitation system of payment which is hoped to replace the fee-for-service approach (National Health Insurance Authority 2012).

Within the context described earlier, this study focuses on assessing antibiotic prescription practices in primary health-care settings in Ghana using the WHO/INRUD (World Health Organization 1993) rational drug use indicators, and exploring for factors influencing practices.

## Study methods and population

### Study settings and population

The study was conducted in four primary health-care facilities in New Juaben Municipality and Atiwa District in the Eastern Region, Ghana between April and July 2011. Facilities were selected purposefully in collaboration with the Health Administration in New Juaben Municipality and Atiwa District and included the main facility types offering general outpatient care. One of the facilities was located in a rural community and three in urban. One institution was a hospital (owned by a not-for-profit religious organization; prescribers were medical officers), two were clinics (one owned by a not-for-profit religious organization and the second a private profit-making institution; prescribers were medical officers and medical assistants in both clinics) and one a health centre (government institution; prescriber was a medical assistant). The urban health facilities had diagnostic laboratories, but none perform microbial culture and drug sensitivity tests. Access to point-of-care tests to differentiate diagnosis of infections was limited to rapid diagnostic tests (RDTs) for malaria and used by the two not-for-profit religious institutions. Test kits were obtained through government sources. The private clinic and the health centre did not use RDT.

To assess antibiotic use practices, a retrospective audit of all outpatient prescriptions (irrespective of patient age and diagnosis) was conducted for the period April 2010 to March 2011. With the level of antibiotic use as the main study outcome of interest, sample size was determined for the smallest detectable effect of 5% (i.e. 5% departure from the 43% estimated by Arhinful 2009), a significance level of 0.05 (two-tailed test) and power of 95% (Cohen 1988). The required sample size was estimated to be 1275, and this was rounded up to 1600 prescriptions. Four hundred patient prescriptions were therefore systematically sampled in each health facility. Based on the average annual outpatient attendance for each facility, a sampling interval was determined; intervals of 181 for the hospital, 165 for the private not-for-profit clinic, 50 for the private for-profit clinic and 24 for the health centre were used. Sampling started with a random pick of the first prescription and the sampling interval was used to select the remaining. Information obtained from prescriptions included patient demographics (age, gender and health insurance status), illness signs and symptoms, diagnostic tests conducted, diagnosis and medicines prescribed. The data were extracted from health facility records using a standardized data collection tool. To ensure confidentially, patients’ names were not recorded, but the record indexing system used by the health facilities were adapted in generating codes that allowed only the research team to be able to link filled tools with the source data.

Three research assistants with post-secondary diploma in community health were engaged. A training session for research assistants was held prior to embarking on data collection and focused on the aim of the study, quality in the data collection approach and ethical considerations. The data collection tool was pre-tested in a polyclinic in Accra. Ten percent of the records were sampled and cross-checked against patient records at health facilities by the primary researcher as a quality control measure.

### Data analysis

Data were entered in Epi-Info software version 3.5.1 and cleaned. Analysis was done using IBM Statistical Package for Social Sciences software version 20. Core prescribing indicators were computed in line with the WHO/INRUD (World Health Organization 1993) rational drug use methodology. Indicators included average number of medicines per prescription; the percentage of patients prescribed (1) an antibiotic and (2) an injection; the percentage of prescribed medicines from the essential medicines list (EML) and the percentage of medicines prescribed by generic name, The International Non-proprietary Names of medicines in the National EML was used in deciding whether a drug is generic or not (Ministry of Health 2010a). In addition, the following products/words—fersolate (ferrous sulphate), vitamin B complex, vitamin C, antacid and cough syrup, when stated on prescriptions, were considered generics. Antibiotics were defined as antibacterial agents including metronidazole, irrespective of formulation. Metronidazole was considered an antibiotic because it is widely used in Ghana for its activity against anaerobic bacteria (Freeman *et al*. 1997). With the exception of immunizations and contraceptives, all injectable products prescribed were considered injections. In the dataset, 11% of diagnoses were stated as respiratory tract infection. To assess rational antibiotic use in this set of cases, this group was re-classified as upper or lower respiratory tract infections (LRTIs). Any symptom or sign stated in patient records suggesting fever (body temperature > 37°C—taken in the mouth or armpit) or chills, breathlessness, wheezing, chest pain, productive cough or a cough lasting 2 weeks or more were classified as LRTIs. All other respiratory infections were classified as URTIs.

Logistic regression was used in data analysis (Machin *et al*. 2007). The outcome variable of interest was an antibiotic prescription. Explanatory variables included in the analysis were type of health facility the patient attended, patient age, gender, health insurance status, number of diagnoses stated by prescriber, number of medicines prescribed and malaria medicine prescription. The relationship between antibiotic prescription and a number of diagnoses (malaria, URTI, dermatological disease and musculoskeletal disease) were investigated. These diagnoses were selected as they were the most common in the sample population. LRTI, though the third most common diagnosis was not included in the statistical analysis because all cases received an antibiotic. Other explanatory variables that may be of interest such as the type of prescriber consulted for each patient encounter, type of diagnostic tests performed on patient, type of consultation (new case or review), presence of fever (measured on a temperature scale) and previous prescriptions and treatment given were not included in the analysis because of data quality issues and the lack of consistency in amount of information stated in patient records across health facilities. Odds ratio (OR) and confidence intervals (95% CIs) for antibiotic prescription across categories of the explanatory variables were calculated. Both bivariate and multivariate analyses were done because of possible interrelationships between explanatory variables and also to adjust for factors that might affect antibiotic prescribing that are non-randomly distributed in comparison groups (Houston and Naylor 1996).

## Results

### Characteristics of outpatients

The characteristics of outpatients, as obtained from prescription records, are presented in Table 1. Out of the 1600 prescriptions, 289 (18.1%) were for children below 5 years, 292 (18.3%) were for children 5 to below 15 years, 890 (55.6%) were for adults 15–64 years and 129 (8.1%) were for persons 65 years and above. Females dominated with a total of 974 patients (60.9%). Most patients (1444; 90.3%) were enrolled under the NHIS

**Table 1.** Patient characteristics in sampled records

Variables	Percentage (%)	% population^a^	*n*_j_/*n*_k_^b^^,^^c^
Patient age (years)			
<5	18.1	13.8	289/1600
5–14	18.3	24.5	292/1600
15–24	16.0	20.0	256/1600
25–44	23.9	25.7	382/1600
45–64	15.8	11.3	252/1600
≥65	8.1	4.7	129/1600
Gender			
Male	39.1	48.8	626/1600
Female	60.9	51.2	974/1600
Health insurance status^a^^,^^d^			
Insured	90.3	35.2	1444/1590
Non-insured	9.1	64.8	146/1590
Diagnosis^e^ (% prescribed antibiotics)			
Malaria	61.8 (54.3)		893/1446
Upper respiratory infection	17.3 (86.4)		250/1446
Lower respiratory infection	11.2 (100)		162/1446
Musculoskeletal disorders	8.4 (24.8)		121/1446
Dermatological diseases	8.0 (84.5)		116/1446
Intestinal worms	6.2 (64.4)		90/1446
Arthritic disorders	6.0 (18.4)		87/1446
Anaemia	5.7 (46.3)		82/1446
Gastroenteritis	5.6 (95.1)		81/1446
Enteric fever	4.1 (100)		60/1446
Others	29.3 (63.5)		463/1446
Medicines prescribed^e^^,^^f^			
Analgesics	27.1		1735/6412
Antibiotics	19.1		1223/6412
Antimalarials	18.1		1160/6412
Vitamins and supplements	13.0		833/6412
Cough preparations	4.7		299/6412
Iron-based haematinics	3.2		208/6412
Others	14.9		954/6412
Number of medicines prescribed			
≤2 medicines	12.2		195/1600
3 medicines	29.4		471/1600
4 medicines	23.5		376/1600
≥5 medicines	34.9		558/1600

^a^Age and gender distribution data from Ghana Statistical Service, 2012; Health insurance coverage data from National Health Insurance Authority, 2012 Annual Report.

^b^*n*_j_ numerator.

^c^*n*_k_ denominator.

^d^No health insurance status was indicated on 10 prescriptions and were excluded in the analysis.

^e^Percentages do not add up to 100 as most prescriptions had >1 diagnoses stated/ medicines prescribed. One hundred and fifty-four prescriptions had no diagnosis stated and were excluded in the analysis.

^f^Denominator for calculation of percentages is number of medicines prescribed.

### Diagnoses

Diagnoses stated in patients’ records were provisional or final. In records where final diagnoses were not stated, provisional diagnoses were used to represent diagnoses. Where no diagnosis was stated, it was noted as such and no attempt was made to use presenting symptoms to assume a diagnosis. One hundred and fifty-four (9.6%) prescriptions had no diagnosis stated and 866 prescriptions had only provisional diagnoses. Malaria and respiratory tract infections were the most common diagnoses representing prescribers’ impression in 61.8% and 28.5% of encounters, respectively (Table 1). Many prescriptions had multiple diagnoses stated and the average number per encounter was 1.69 (Table 2).

**Table 2.** Prescribing indicators in primary health facilities in Eastern Region, Ghana

Prescribing indicators (World Health Organization, 1993)	All	Hospital	Health centre/clinic
Average number of medicines prescribed per encounter (SD)	4.01 (1.406)	3.27 (1.140)	4.25 (1.400)
% of prescriptions with antibiotics	59.9	48.2	63.7
% of medicines prescribed by generic name	79.2	61.4	83.7
% of encounters with an injection prescribed	22.9	19.8	24.0
% of medicines prescribed from EML	88.1	78.3	90.6
% of prescriptions with drug combinations known to interact	4.9	2.5	5.7
Average number of diagnosis per clinical encounter (SD)	1.69 (0.739)	1.44 (0.600)	1.78 (0.765)

SD, standard deviation; EML, essential medicines list.

### Medicine prescription practices

Antibiotics were the second most commonly prescribed medicines (19.1%) after analgesics (27.1%). Other commonly prescribed medicines were antimalarials (18.1%), vitamins and supplements (13.0%), cough preparations (4.7%) and iron-based haematinics (3.2%).

Antibiotics commonly prescribed were amoxicillin (26.7%), metronidazole (18.2%), flucloxacillin (8.9%), amoxicillin/clavulanic acid (8.2%), ciprofloxacin (8.2%), erythromycin (4.7%), chloramphenicol (3.4%) and co-trimoxazole (2.8%). All the antibiotic products listed earlier were for systemic use. Antibiotics for dermatological application formed 6.0% of antibiotics used and most products contained the aminoglycoside gentamycin or neomycin, a glucocortico-steroid and an antifungal. Other topical antibiotics formed 3.9% of prescribed antibiotics. Only 4.2% of prescribed antibiotics were given as injections.

Only two clinical encounters led to no drugs being prescribed: one patient detected to be pregnant and the other with a valgus deformity (an abnormal outward turning of the distal segment of a bone or joint). A summary of prescribing and quality care indicators are presented in Table 2. The average number of medicines prescribed per encounter was 4 and 59.9% of clinical encounters led to an antibiotic (antibacterials and metronidazole) prescription. Antibacterials (excluding metronidazole) were prescribed in 53.8% of clinical encounters and metronidazole in 13.7%. Health centre/clinics prescribed more drugs per clinical encounter compared with the hospital (4.25 vs 3.27; *P*-value < 0.001, 95% CI: 0.84, 1.14) and prescribed antibiotics more often (63.7 vs 48.2%; *P*-value < 0.001, 95% CI: 1.50, 2.37). Approximately 5% (4.9%) of all prescriptions included drug combinations with known interactions. Most interactions were between artemether-lumefantrine and the quinolones, or macrolides, or ketoconazole, or fluconazole, drug combinations advised against by the manufacturer of artemether-lumefantrine (Joint Formulary Committee 2009).

The outcomes of the logistic regression are presented in Table 3; the bivariate analysis output is in the left panel and the multivariate (adjusted odds) in the right panel. In the multivariate analysis, health facility type, patient age, number of medicines prescribed and an antimalarial drug prescription were significantly associated with an antibiotic prescription. The diagnosis stated by the prescriber for patients’ symptoms may also influence antibiotic prescribing. In the multivariate analysis, the three variables—malaria diagnosis, URTI diagnosis and musculoskeletal disorder diagnosis—each independently and significantly were associated with an antibiotic prescription. Attending a health centre/clinic vs a hospital increased the odds of an antibiotic prescription (OR = 2.05, 95% CI: 1.42, 2.95). Being of a young age greatly increased the possibility of an antibiotic prescription. In total, 77.5% of children below 5 years, 71.6% of the age group 5–14 years, 53.5% of those 15–64 years and 38% of the elderly 65 years and above received an antibiotic. The odds of getting an antibiotic prescribed decreased by 3% for every 1 year increase in age (OR = 0.97; 95% CI: 0.97, 0.98). As the number of medicines prescribed per encounter increased by one, the odds of an antibiotic prescription increased by 85% (OR: 1.85; 95% CI: 1.63, 2.1). Having an antimalaria drug on a prescription was protective against an antibiotic prescription. Prescriptions without antimalaria drugs were five times more likely to have an antibiotic when compared with prescriptions with an antimalarial (OR = 5.05; 95% CI: 2.08, 12.25). Being diagnosed with ‘malaria only’ was protective against an antibiotic prescription. A ‘no malaria’ diagnosis increased the odds of an antibiotic prescription by 4.72 (95% CI: 1.86, 11.99) compared with a ‘malaria only’ diagnosis. A diagnosis of an URTI, either alone (OR = 7.27; 95% CI: 1.66, 31.82) or with other concomitant conditions (OR = 1.86; 95% CI: 1.18, 2.93) increased the odds of an antibiotic prescription compared with when there was no diagnosis of URTI.

**Table 3.** Factors influencing antibiotic prescribing practice

Independent variables	Bivariate analysis				Multivariate analysis			
	*N*	OR	95% CI	*P*-value	*n*	OR	95% CI	*P*-value
Type of health facility								
Hospital^a^	400	1			390	1		
Health centre/clinic	1200	1.89	1.50–2.37	<0.0001	1046	2.05	1.42–2.95	<0.0001
Patient age^b^ (years)	1600	0.98	0.97–0.98	<0.0001	1436	0.97	0.97–0.98	<0.0001
Gender								
Female^a^	974	1			880	1		
Male	626	1.29	1.05–1.59	0.015	556	1.17	0.88–1.56	
Health insurance status								
Insured^a^	1444	1			1296	1		
Non-insured	146	1.08	0.76–1.53	0.682	140	0.78	0.49–1.23	0.279
No. of diagnoses stated								
1 diagnosis^a^	654	1			651	1		
≥2 diagnoses	792	3.82	3.06–4.76	<0.0001	785	1.45	0.85–2.47	0.172
No. of medicines prescribed^b^	1600	1.83	1.68–2.01	<0.0001	1436	1.85	1.63–2.10	<0.0001
Malaria treatment prescribed								
Yes^a^	993	1			867	1		
No	607	1.47	1.19–1.82	<0.0001	569	5.05		<0.0001
Malaria diagnosis								
Malaria only^a^	290	1			289	1		
No malaria	707	10.50	7.37–14.82	<0.0001	551	4.72	1.86–11.99	0.001
Malaria + other condition(s)	603	13.73	9.58–19.67	<0.0001	596	4.95	2.62–9.37	<0.0001
URTI diagnosis								
No URTI^a^	1350	1			1187	1		
URTI only	42	16.40	3.95–68.08	<0.0001	42	7.27	1.66–31.82	0.008
URTI + other condition(s)	208	4.51	3.05–6.67	<0.0001	207	1.86	1.18–2.93	0.008
Dermatological disease diagnosis								
No dermatological disease^a^	1484	1			1320	1		
Dermatological disease	26	3.05	1.14–8.13	0.026	26	1.95	0.64–5.97	0.241
Dermatological disease + other condition(s)	90	4.30	2.37–7.81	<0.0001	90	1.56	0.80–3.06	0.197
Musculoskeletal disorder diagnosis								
Musculoskeletal disorder only^a^	61	1			61	1		
No musculoskeletal disorder	1479	18.90	7.51–47.37	<0.0001	1315	20.40	7.57–55.00	<0.0001
Musculoskeletal disorder + other condition(s)	60	8.00	2.80–22.84	<0.0001	60	6.89	2.14–22.22	0.001

*n,* number of prescriptions; OR, odds ratio; CI, confidence interval.

^a^Reference value.

^b^Continuous variable.

## Discussion

This study presents the results of a prescription practice audit in primary health-care facilities in Eastern Region, Ghana. The WHO/INRUD (World Health Organization 1993) rational drug methodology was used. In the descriptive analysis, the average number of medicines prescribed per prescription, the proportion of prescriptions with an antibiotic, the proportion prescribed an injection and the use of generic name of drugs in prescribing were high (4.0, 59.9%, 22.9% and 79.2%, respectively) compared with estimates of 3.6, 43.3%, 13.3% and 59.9%, respectively, reported in a survey conducted by the MOH, Ghana in 2008 (Arhinful 2009). This study, when compared with the MOH study, is smaller with respect to the number of health facilities and prescriptions sampled, but have its strength in the fact that most prescriptions (75 vs 23.4% in MOH study) were from lower level health facilities that constitute >80% of health service delivery points in Ghana (Ghana Health Service 2010). The types of health facilities studied and prescribers at each level of care may influence the study outcome as prescribing practices of physicians are known to differ from non-physician prescribers (Holloway *et al*. 2013). In Ghana, physicians are more common in hospitals than in lower-level facilities, and skewing data collection towards hospitals may lead to findings mirroring practices at this level of care. Primary health centres/clinics in Ghana serve as first points of contact with formal health services (Zwart and Voorhoeve 1990; Bour 2003). Since the referral systems in Ghana encourage treatment of acute infections at lower-level facilities and referral of complications and chronic conditions such as cardiovascular diseases to hospitals (Ministry of Health 2010b), primary health-care facilities are more likely to have clients with acute infectious diseases. Therefore, a higher proportion of prescriptions with antibiotics may be expected compared with prescriptions from hospital outpatient settings. This may partly explain the level of antibiotic use in this study, but compared with the disease profile of Ghana, the use level is considerable high. Another plausible cause of high antibiotic use is diagnostic uncertainty since most prescriptions had only provisional diagnoses stated and clinicians may prescribe liberally, including the use of antibiotics, to cover all the possible causes of symptoms. Regarding the increased use of medicines among patients, the drug reimbursement method under Ghana’s NHIS could be a contributory cause (Witter and Garshong 2009). Compared with 2008, the proportion of patients accessing formal health-care services that are insured has increased (Ghana Health Service 2012a). With free drugs promised under the NHIS (>500 medicine formulations on the NHIS list), drug prescribing staff may be less conscious of cost to be incurred by patient and this would favour polypharmacy (Williams *et al*. 2012). The fee-for-service approach in reimbursing drug cost may be an additional incentive to practise polypharmacy (Hulscher *et al*. 2010).

The study findings suggest a higher use of medicines at lower health facilities and a generally high antibiotic use compared with the average reported in the MOH study (Arhinful 2009). Further research is required to know whether rational drug use is worsening in Ghana at the national level. A review that comprised 900 studies conducted between 1990 and 2009 in primary health-care settings in 104 developing and transition countries concluded that medicine use is not improving over time (Holloway *et al*. 2013). The median number of medicines prescribed per encounter increased from 2.1 (before 1992) to 2.8 in 2007–09, whilst the proportion of patients receiving an antibiotic increased from 42 to 51% over the same period. Level of injection use was reported as stable around 20% in the period. These findings suggest a need for targeted interventions beyond the efforts of the last three and a half decades of a WHO-led country-implemented rational drug use strategies. The WHO essential medicines concept for example have improved access to medicines in many countries (Laing *et al*. 2003), but its effect on prudent use of medicines remains unclear (Ratanawijitrasin *et al*. 2001).

The logistic regression model considered the association between a set of explanatory variables and an antibiotic prescription. Ghana Health Service (2010) reports suggest that, excluding malaria and intestinal worms, other infectious diseases constitute ∼20% of morbidity in outpatients, but there is no information on disease patterns across age groups. Even though ∼33% of diagnoses arrived at by clinicians for patients symptoms suggest an infection (excludes malaria and intestinal worms), the level of antibiotic prescriptions recorded in the study must still be considered high. In children below 5 years, 77.5% were prescribed an antibiotic. In Ghana, mortality rates in this age group, though reducing, is still high (80 per 1000 live births) and may be influencing prescribing decisions to err on the side of caution when unsure of underlining cause of symptoms (Ghana Statistical Service 2009). What was more surprising was the level of antibiotic prescribing in the age group 5–14 years which was equally high. Burden of disease in children in this age group is expected to be low compared with below 5-year–olds, and children in this group are less at risk of life-threatening infectious diseases (Würthwein *et al*. 2001; West 2002; Murray *et al*. 2012). The high level of antibiotic use in this age group could be partly explained by the significantly lower health service utilization rate (*P*-value < 0.001; 95% CI: 16.4%, 20.2%), which suggests a selection for severely ill children in seeking care.

Insurance systems that incorporate co-payments for drug prescriptions are reported to be associated with reductions in the use of essential medicines, including antibiotics (Foxman *et al*. 1987; Harris *et al*. 1990). This study did not find any association between health insurance status of patients and antibiotic use. Foxman *et al*. (1987) showed that the main effect of co-payments on antibiotic use was not a reduction in antibiotic prescription per clinical encounter but rather a reduction in health service utilization resulting in a significantly lower number of antibiotic prescriptions for persons on co-payment plans compared with patients with full insurance coverage. This study was not designed to detect this outcome but an effect of insurance on health service utilization can be expected considering the difference in insurance coverage among outpatients compared with the general population of Ghana (Ghana Health Service 2012a; National Health Insurance Authority n.d). Health insurance could however have an indirect effect on prescription practices. In both public and private health-care facilities, revenue generated from services is the main source of income for financing recurrent expenditure including salaries in some cases (Ghana Health Service 2004, 2012b). With standardized insurance reimbursement rates, health facility income generation is dependent on their ability to retain clients. Since antibiotics are viewed by both health professionals and the public as powerful medicines, there may be a tendency to over-prescribe these which reinforce existing perceptions and keep clients satisfied (Radyowijati and Haak 2003). Second, antibiotics are high-value commodities with high margin of return for health facilities when prescribed (World Bank 2009). This may serve as an additional motivation to prescribe antibiotics, especially when clinicians have been reported to have the notion that antibiotics, if not useful, are not harmful (Amábile-Cuevas 2010). In Ghana, it is known that patients often equate medicines with health care (Ministry of Health 2004), and the characteristics of medicines prescribed such as the quantity may signal the quality of care. Therefore, it is not surprising that the practice of polypharmacy was present in the study settings. And as the number of medicines prescribed increased, the odds of receiving an antibiotic also increased. Interventions to reduce the number of medicines prescribed per encounter should take cognizance of the positive association between polypharmacy and antibiotic prescribing rates to be able to address both challenges. As the main purchaser of health-care services in Ghana, the NHIS is strategically positioned to introduce systems that can modernize health-care delivery in Ghana (Schieber *et al*. 2012) and this potential, with the help of stakeholders, must be exploited in support of rational drug use.

In reducing medicine use and antibiotic prescription rate, special attention has to be given to the diagnosis and treatment of malaria and acute respiratory infections, the two most common diagnoses in the study settings and both significantly associated with antibiotic use. Diagnostic tools that help clinicians to narrow their suspicions to arrive at definite diagnoses may be useful and may impact on antibiotic prescribing since many illnesses presenting in health facilities do not have a bacterial cause (Petit *et al*. 1995; Schwarz *et al*. 2010; Kwofie *et al*. 2012). A limitation however is whether prescribers would take advantage of available diagnostic services or tools. Studies have shown that this does not always happen (Mohan *et al*. 2004; Polage *et al*. 2006). In this study, it was observed that narrowing a diagnosis to a non-bacterial cause such as malaria still led to antibiotic prescriptions in some cases; 16.2% of malaria only cases received antibiotics. Rapid diagnostic testing for malaria is currently promoted in many developing countries including Ghana, and there is the possibility of increasing the proportion of patients with non-malaria fevers that may end up being treated blindly with antibiotics (Msellem *et al*. 2009; Mosha *et al*. 2010). Point-of-care tests to differentiate fever of bacterial origin from other causes of fever is therefore necessary and has been shown to significantly reduce antibiotic prescribing (Bjerrum *et al*. 2006; Llor *et al*. 2012). Kwofie *et al*. (2012) observed in a tertiary hospital in Ghana that, out of a sample of 128 children 5 years and below presenting with acute LRTI, only 12 (9.4%; 95% CI: 4.9%, 15.8%) had a bacterial infection. Petit *et al*. (1995), in a study conducted in Ghana and Kenya involving 639 in-patients with fever, reported that only 11.3% of patients had a bacterial infection. These studies further highlight the importance of point-of-care diagnostic testing in promoting rational antibiotic use and improving quality of care. While we can expect that health services, including diagnostic capacities at health facilities, will improve with increasing economic growth of a country, barriers to the use of existing diagnostic tools such as RDT for malaria and illuminators for throat examinations that can reduce diagnostic uncertainty and improve antibiotic use must be examined.

## Study limitations

The study adopted a retrospective data collection approach that reduces the possibility of Hawthorne effect (i.e. clinician awareness that they are being observed). However, the depth and quality of data available at health facilities influenced the variables that could be studied. Variables such as diagnostic tests performed and outcomes, signs of infection such as fever, prescriber type and characteristics, patient load and previous treatment may influence antibiotic prescribing behaviour, but were not studied because of limitations of data (Radyowijati and Haak 2003; Bjerrum *et al*. 2006; Das and Das 2006). The usefulness of rational drug use studies as a quality of care monitoring mechanism may therefore be limited unless the scope and depth of information that clinicians document as part of consultations is examined and standards introduced. Prescriptions were drawn from only four health facilities (one health centre, two clinics and one hospital) representing the various types of facilities in primary care. Differences observed in prescribing practice across health facility types should therefore not be generalized.

## Conclusion

The level of antibiotic use in primary health-care facilities in the study settings was observed to be high when compared with earlier estimates for Ghana as well as the average reported for developing and transition countries (Arhinful 2009; Holloway *et al*. 2013). Comparing the level of antibiotic use with the disease profile of Ghana (Ghana Health Service 2010), inappropriate use is probable. A reassessment of rational drug use indicators at the national level in Ghana is overdue (Laing *et al*. 2001). This study suggests that the level of antibiotic use across health facility types is not uniform and a representative sampling strategy that considers the diversity in health facility types in Ghana is therefore necessary in future national surveys in estimating antibiotic use.

Of more importance are the interventions that countries such as Ghana with high levels of antibiotic use are able to put in place to promote rational use. Despite the limitations of this study, a clear significant association has been demonstrated between antibiotic use and malaria and respiratory tract infections, the two common diagnoses in primary health care. Interventions must therefore target the diagnosis and management of these conditions. From literature, several intervention options are available to address inappropriate antibiotic use and research into methods suitable for the study settings is needed.
